# A Web-Based Human Papillomavirus Vaccination Intervention for Young Gay, Bisexual, and Other Men Who Have Sex With Men: Protocol for a Randomized Controlled Trial

**DOI:** 10.2196/16294

**Published:** 2020-02-24

**Authors:** Paul L Reiter, Amy L Gower, Dale E Kiss, Molly A Malone, Mira L Katz, Jose A Bauermeister, Abigail B Shoben, Electra D Paskett, Annie-Laurie McRee

**Affiliations:** 1 The Ohio State University College of Public Health Columbus, OH United States; 2 The Ohio State University Comprehensive Cancer Center Columbus, OH United States; 3 Division of General Pediatrics and Adolescent Health, University of Minnesota Medical School Minneapolis, MN United States; 4 Department of Family and Community Health, University of Pennsylvania School of Nursing Philadelphia, PA United States; 5 The Ohio State University College of Medicine Columbus, OH United States

**Keywords:** human papillomavirus, human papillomavirus vaccination, gay or bisexual, men who have sex with men, intervention, young adult

## Abstract

**Background:**

Gay, bisexual, and other men who have sex with men experience several disparities related to human papillomavirus (HPV) infection, including high incidence rates of anal cancer. Although the HPV vaccine is currently recommended for young adults, HPV vaccine coverage is modest among young gay, bisexual, and other men who have sex with men (YGBMSM).

**Objective:**

We describe the design and methods for a randomized controlled trial (RCT) to rigorously evaluate *Outsmart HPV*, a population-targeted, individually tailored, Web-based HPV vaccination intervention for YGBMSM. The RCT is designed to determine the efficacy of the intervention, the mechanism by which the intervention has an effect (ie, mediation), and whether efficacy varies by participant characteristics (ie, moderation).

**Methods:**

*Outsmart HPV* was previously developed and pilot-tested. This study is a 3-arm prospective RCT that will enroll a projected 1995 YGBMSM who are aged 18 to 25 years, live in the United States, and have not received any doses of the HPV vaccine. Participants will be recruited by means of paid advertisements on social media sites and randomized to receive (1) standard information on the Web about HPV vaccine (control group), (2) *Outsmart HPV* content on the Web with monthly unidirectional vaccination reminders sent via text messages, or (3) *Outsmart HPV* content on the Web with monthly interactive vaccination reminders sent via text messages. Participants will complete Web-based surveys at 4 time points during the study: baseline, immediately after engaging with Web-based content, 3 months after randomization, and 9 months after randomization. Primary outcomes will include both HPV vaccine initiation (ie, receipt of 1 or more doses of the HPV vaccine) and completion (receipt of all 3 doses recommended for this age range). We will examine constructs from the intervention’s theoretical framework as potential mediators and demographic and health-related characteristics as potential moderators of intervention effects.

**Results:**

The institutional review board at The Ohio State University has approved the study. Materials have been developed and finalized for all study groups. Recruitment for the RCT began in fall 2019.

**Conclusions:**

If shown to be efficacious, *Outsmart HPV* has the potential to fill an important gap by promoting HPV vaccination among a population at increased risk of HPV infection and HPV-related disease.

**Trial Registration:**

ClinicalTrials.gov NCT04032106; http://clinicaltrials.gov/show/NCT04032106

**International Registered Report Identifier (IRRID):**

PRR1-10.2196/16294

## Introduction

About 45% of men in the United States have a current genital infection with at least one type of human papillomavirus (HPV) [1]. Infection with oncogenic HPV types can cause several types of cancer among men (eg, anal, oropharyngeal, and penile cancers), and infection with nononcogenic types can cause genital warts [2,3]. Several HPV-related disparities exist among gay, bisexual, and other men who have sex with men (GBMSM). Past research has shown that up to about 66% of GBMSM who are HIV negative have a current genital HPV infection, with prevalence estimates being even higher among those who are HIV positive [4]. Anal cancer incidence rates among GBMSM have ranged to more than 60 cases per 100,000 population, which is much higher than the national rate of about 2 cases per 100,000 population for men [5-9].

Although the Advisory Committee on Immunization Practices (ACIP) began recommending routine HPV vaccination for females in the United States in 2006 [10], routine vaccination was not recommended for males until 2011 [11]. The ACIP now recommends routine HPV vaccination for both male and female adolescents aged 11 to 12 years and recently voted unanimously to support catch-up vaccination for persons through the age of 26 years who are not vaccinated [12]. Before this recent update, catch-up vaccination was recommended only for males aged 13 to 21 years, although routine HPV vaccination was still recommended for men who have sex with men (including those who identify as gay or bisexual or who intend to have sex with men) through the age of 26 years [13]. The recent update also now recommends that adults aged 27 to 45 years make shared decisions with their doctors about getting the HPV vaccine [12]. The HPV vaccine series consists of 2 doses if it is initiated before the age of 15 years and 3 doses if it is initiated after the age of 15 years [13]. HPV vaccination is currently approved to prevent anal cancer and genital warts among males [14].

Despite recommendations, HPV vaccine coverage is modest among young gay, bisexual, and other men who have sex with men (YGBMSM) in the United States. Studies have shown that about half or fewer YGBMSM have received any HPV vaccine doses, and fewer than 20% have received all recommended doses [15-22]. Although HPV vaccine coverage may be slighter higher among GBMSM than among heterosexual men [16,23], the suboptimal coverage is still somewhat surprising, given that most GBMSM have expressed a willingness to get vaccinated [24-28]. YGBMSM have reported numerous barriers to HPV vaccination, including lack of knowledge about HPV and the HPV vaccine, concerns about vaccine safety, concerns about cost and/or health insurance, perceived stigma around the vaccine (eg, the vaccine is only for females), fear of potential health care discrimination, and lack of a health care provider recommendation to get vaccinated [17,20,29]. Given that many YGBMSM remain unvaccinated, efforts to increase HPV vaccine coverage among these men are needed.

We recently developed and pilot-tested a Web-based HPV vaccination intervention for YGBMSM called *Outsmart HPV* [30]. During the pilot test, we recruited 150 unvaccinated YGBMSM from the United States through social media [31]. We randomized participants to receive either standard information about the HPV vaccine (control group) or population-targeted, individually tailored content about the HPV vaccine and monthly unidirectional vaccination reminders (*Outsmart HPV* intervention group). The results of the pilot test showed that HPV vaccine initiation was higher among participants in the intervention group (34/76, 45%) than those in the control group (19/74, 26%; *P*=.02) [30]. We also observed a trend toward HPV vaccine completion being higher among the intervention group (8/76, 11%) than among the control group (2/74, 3%; *P*=.07) [30], and the intervention group reported larger changes in several attitudes and beliefs about HPV and the HPV vaccine [32]. Participants in the intervention group reported high levels of acceptability and satisfaction with *Outsmart HPV* [32].

The next step in this line of research is to implement a well-powered randomized controlled trial (RCT) to determine the efficacy of *Outsmart HPV*. It is important to also identify the mechanism by which the intervention has an effect (ie, mediation) and determine how efficacy may vary by participant characteristics (ie, moderation). This study describes the protocol for a study that will accomplish this by comprehensively evaluating *Outsmart HPV*.

## Methods

### Trial Overview

**Figure 1:**
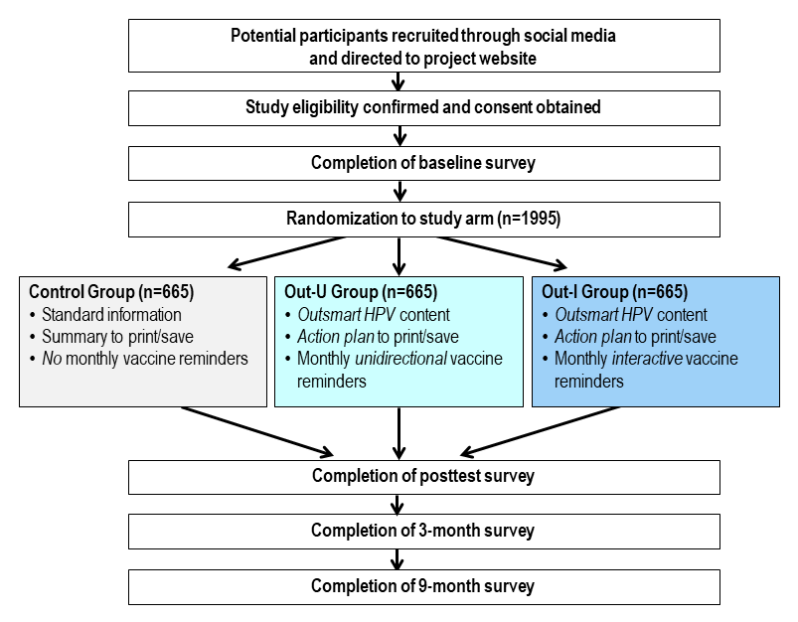
Overview of the randomized controlled trial for the *Outsmart HPV* intervention. HPV: human papillomavirus; Out-I group: *Outsmart HPV* that includes monthly interactive vaccination reminders; Out-U group: *Outsmart HPV* that includes monthly unidirectional vaccination reminders.

This study is a 3-arm prospective RCT that will enroll a projected 1995 YGBMSM (Figure 1). Participants will be randomized to receive (1) standard information about the HPV vaccine (control group), (2) *Outsmart HPV* with monthly unidirectional vaccination reminders (Out-U group), or (3) *Outsmart HPV* with monthly interactive vaccination reminders (Out-I group). Each group is described in detail in the following sections. We will follow up with the participants for 9 months and use an intent-to-treat approach to determine the effects of *Outsmart HPV* on HPV vaccination.

### Recruitment and Eligibility

We will recruit YGBMSM through paid advertisements on social media sites. Social media sites will include Facebook and Instagram, with the potential for expanding to other sites if needed. About 90% of adults aged 18 to 29 years in the United States use social media, with Facebook and Instagram being 2 of the most popular platforms for these ages [33,34]. For the pilot test, we also recruited by means of social media advertisements, and it resulted in a sample of participants who were demographically similar to nationally representative samples of YGBMSM [31].

Advertisements will link potential participants to the project website, which is a mobile-friendly website accessible by desktop, laptop, tablet, or smartphone (iOS and Android). Eligibility will be based on responses to a Web-based screener. Potential participants will be eligible if they (1) are cisgender male; (2) are aged between 18 and 25 years; (3) self-identify as gay, bisexual, or queer; report ever having oral or anal sex with a male; or report being sexually attracted to males; (4) live in the United States; (5) have not received any doses of the HPV vaccine; and (6) did not participate in the pilot test of *Outsmart HPV*. We specify the age of 25 years instead of 26 years as the upper age limit so that participants do not *age out* of the recommended age range for routine vaccination [12,13] during the study. We will also require men to read English and be able to provide informed consent, both of which will be inferred by completion of the eligibility screener and consent form. After completing the screener, eligible men will provide informed consent and create a project account (eg, establishing a username and password and providing an email address and text message number to allow for study communications).

### Initial Website Session and Randomization

After account setup, participants will begin their initial project website session. This will consist of completing a baseline survey on the Web, engaging with Web-based content about the HPV vaccine (either *Outsmart HPV* or control group materials), and then completing a posttest survey on the Web. Immediately after completing the baseline survey, participants will be randomized using a 1:1:1 allocation scheme to receive (1) standard information about the HPV vaccine (control group), (2) *Outsmart HPV* that includes monthly unidirectional vaccination reminders (Out-U group), or (3) *Outsmart HPV* that includes monthly interactive vaccination reminders (Out-I group). Recruitment will continue until a projected 1995 YGBMSM are randomized (about 665 per study group).

### Study Materials

Materials for all study groups are patterned heavily after those from our pilot test [30,32]. To further help ensure the appropriateness of study materials, we conducted focus groups of YGBMSM before the RCT to review and discuss study materials. The project website will deliver materials to all study groups.

#### Outsmart HPV With Monthly Unidirectional Vaccination Reminders

**Figure 2:**
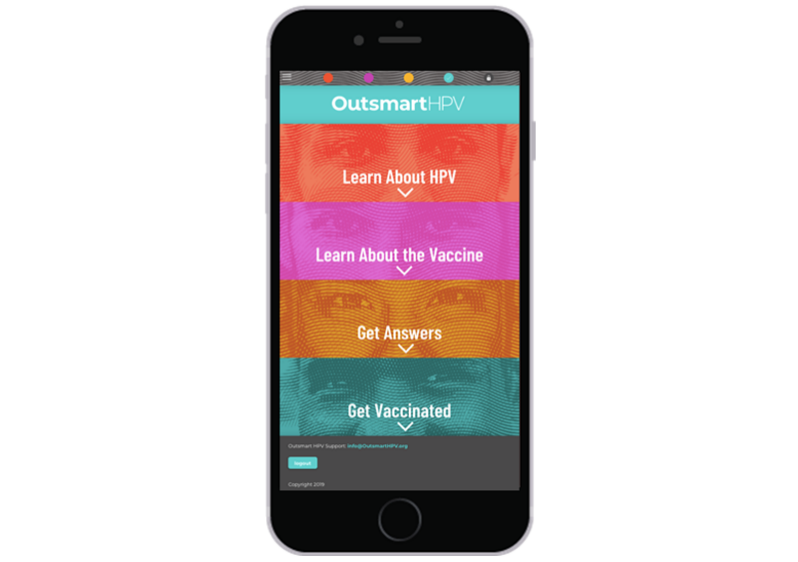
Screenshot from a smartphone of the *Outsmart HPV* intervention showing the 4 sequential sections. Both intervention arms (*Outsmart HPV* that includes monthly interactive vaccination reminders and *Outsmart HPV* that includes monthly unidirectional vaccination reminders groups) will receive this content.

**Figure 3:**
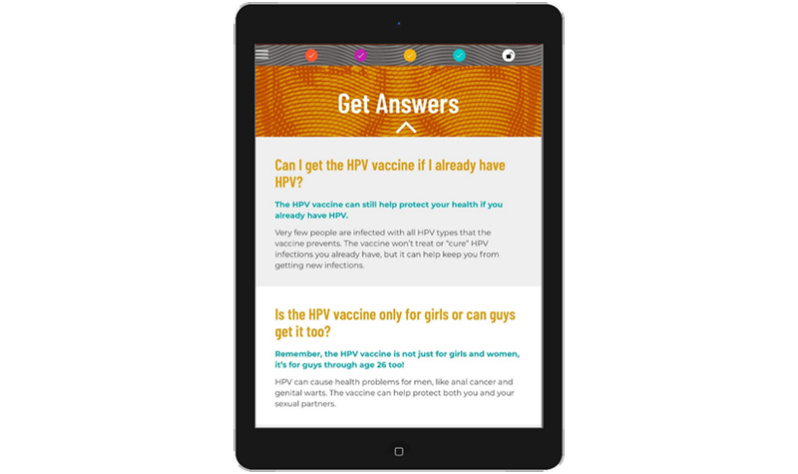
Screenshot from a tablet computer of the Get Answers section of *Outsmart HPV*. Presentation of content is individually tailored to prioritize participants’ barriers and concerns about the human papillomavirus vaccine, as indicated on the baseline survey.

**Figure 4:**
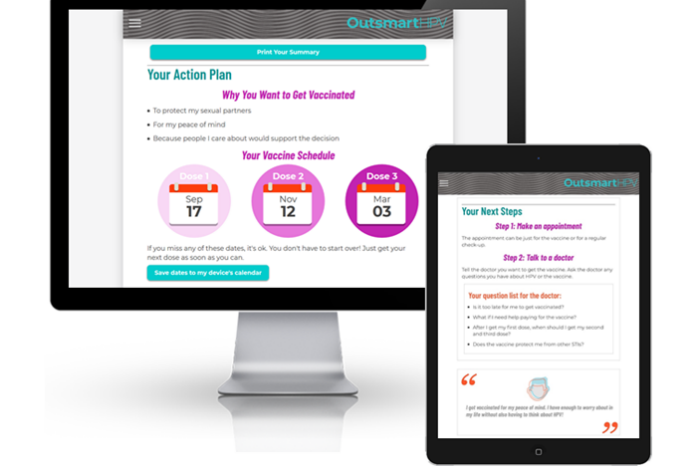
*Outsmart HPV* Action Plan on a desktop computer and a tablet computer. The plan is informed by participants’ engagement with the project website and includes a goal date for getting the first dose of the human papillomavirus vaccine series, schedule for subsequent doses, next steps for getting vaccinated, and tailored information for taking these next steps. Participants may revisit the plan on the study website or save/print a copy for future reference.

*Outsmart HPV* was developed using a framework that included aspects of the Protection Motivation Theory [35], Information-Motivation-Behavioral Skills Model [36], and the Minority Stress Model [37]. The intervention for the Out-U group will consist of 2 components: (1) population-targeted, individually tailored *Outsmart HPV* content on the Web and (2) unidirectional monthly HPV vaccination reminders sent via text messages. The Web-based content will be presented in 4 sequential sections (Figure 2) that contain infographics, other visual formats, and tailored testimonials. The 4 sections are as follows:

1. *Learn About HPV* provides population-targeted information about HPV prevalence, transmission, and HPV-related diseases among GBMSM. For example, this section provides information about the occurrence of anal cancer among GBMSM. At the end of this section, participants are asked to identify what they think is the most important thing they learned about HPV.
2. *Learn About the Vaccine* provides information about the HPV vaccine, including its dosing schedule, recommendations for administration, effectiveness, and safety. This section also includes population-targeted information about the HPV vaccine among YGBMSM (eg, the acceptability of the vaccine reported by YGBMSM in past research) [17]. The end of this section then prompts participants to identify their motivations for wanting to get the HPV vaccine.
3. *Get Answers* uses a question-and-answer format to address barriers and concerns about HPV vaccination (Figure 3). The barriers and concerns include those that have been commonly reported by YGBMSM in past studies [17,20,24,29,38,39]. The website uses information from the baseline survey to prioritize and individually tailor the presentation of content in this section. For example, the potential barriers and concerns indicated by a participant on the baseline survey will appear at the top of this section to highlight this salient content. Following this prioritization, participants will be able to access all remaining questions and answers.
4. *Get Vaccinated* provides content about the logistics of getting the vaccine. This includes resources for accessing the HPV vaccine (eg, a weblink to a lesbian, gay, bisexual, transgender, and queer–friendly provider directory [40]), information about vaccine cost and health insurance, and strategies for talking with a provider about getting vaccinated. This section also asks participants to identify potential questions they have for a doctor about the HPV vaccine. The website then prompts participants to create a customized Action Plan (Figure 4). The plan includes a goal date for getting their first dose, schedule for subsequent doses, next steps for getting vaccinated, and individually tailored information for taking these next steps (eg, resources addressing potential barriers and concerns and potential questions to ask a doctor). It also includes brief information about the HPV vaccine and participants’ motivations for wanting to get vaccinated.

The monthly vaccination reminders for the Out-U group will be unidirectional, meaning that participants will not have the option to respond to the reminders. The unidirectional reminders will be sent via an automated process and contain only text. The text will provide information about HPV and the HPV vaccine, steps for getting vaccinated (eg, making an appointment with a health care provider), and content addressing common barriers and concerns about the HPV vaccine. After completing their posttest survey, participants will have the ability to print or save a version of their *Action Plan*. Participants will also be able to log back into the project website and review the Web-based content throughout the study, except when completing surveys.

#### Outsmart HPV With Monthly Interactive Vaccination Reminders

The intervention for the Out-I group will consist of 2 components: (1) population-targeted, individually tailored *Outsmart HPV* content on the Web and (2) interactive monthly HPV vaccination reminders sent via text messages. Thus, the interventions for the Out-I and Out-U groups will be identical except in the type of monthly vaccination reminders sent to participants: participants in the Out-I group will have the option to respond to and/or ask questions to obtain additional tailored information and resources. Research suggests that interactive reminders may be more preferable and effective than unidirectional reminders [41,42], which could be partly because of their ability to obtain additional information/resources and increased participant engagement. Communications for the interactive reminders will be sent through a combination of automated and manual processes, as described further below. To increase participant engagement, some of the interactive reminder text messages will also contain a meme or brief animation in Graphics Interchange Format (GIF). Similar to the approach taken in other technology-based interventions with adolescents and young adults [43], the memes and GIFs are intended to be funny or motivational and reinforce behavior. The text portion of the messages will focus on areas similar to those described above for the Out-U group.

**Figure 5:**
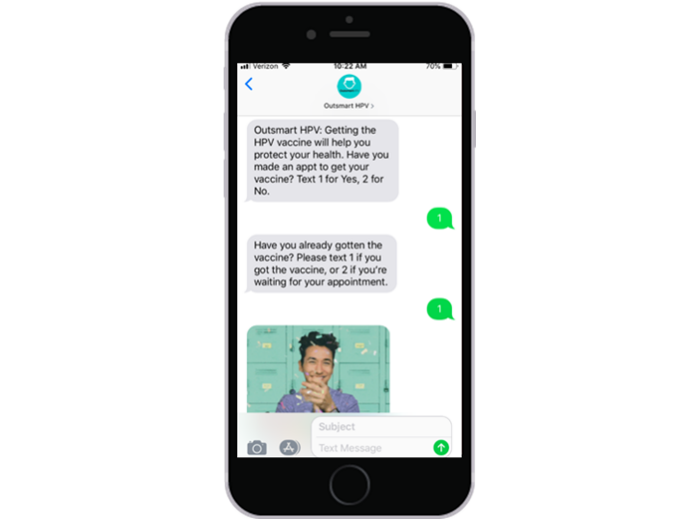
Example of an interactive human papillomavirus vaccination reminder that includes an initial prompted message and subsequent communications. As shown, some of the communications will contain a meme or brief animation in Graphics Interchange Format.

For each month, the initial text message sent to participants will be automated and include a question that prompts a response (Figure 5). Including a prompted message can increase participant responsivity compared with texts with unprompted messages [44]. Depending on how a participant responds to this initial communication, additional automated communications with information and resources will be sent to participants. Participants will then have the opportunity to text further open-ended questions that they may have to the study team (participants can ask as many questions as needed). A study team member will review the received questions and manually send an appropriate response based on a library of example responses that has been developed for this study. Responses to unanticipated questions not included in the library will be discussed and agreed upon by members of the study team. Participants will be notified that it could take up to a few days for the study team to respond to questions.

#### Control Group

Participants in the control group will receive standard information about the HPV vaccine that is closely modeled after the Vaccine Information Statement (VIS) for the HPV vaccine. The VIS is created by the Centers for Disease Control and Prevention to provide easy-to-understand information about the HPV vaccine that is publicly available [45]. We modeled the control group content after the VIS because health care providers are required to give a copy of the VIS to patients before vaccination [45]. We formatted the VIS content to match the color and font scheme of the project website. Similar to the Out-U and Out-I groups, participants in the control group will have the ability to print or save a summary of their viewed content after completing their posttest survey, and they will be able to log back into the project website and review their materials throughout the study (except when completing surveys).

### Follow-Up Surveys and Medical Record Release

After the initial project website session, participants will be asked to complete follow-up surveys 3 and 9 months after randomization, for a total of 4 surveys during the course of the project. The final survey will occur 9 months after randomization to allow participants ample time to receive all 3 doses of the HPV vaccine during the study period. To improve retention, participants in all study groups will be sent automated email and text message notifications to complete their surveys.

Participants who indicate they have received 1 or more doses of the HPV vaccine on the 3- or 9-month survey will be asked to complete a medical record release (MRR) form on the Web. Completion of the MRR is optional; participants who do not complete one will still continue in the study. At the conclusion of the study, the study team will contact the health care providers of participants who complete an MRR form to verify their HPV vaccination status. No additional health information beyond HPV vaccination status will be requested from health care providers, which is indicated to participants during the MRR form process.

### Incentives

Participants will be able to earn up to US $95 in gift cards for this study. This includes a US $40 gift card for completing the initial project website session (ie, baseline survey, engaging with *Outsmart HPV* or control group materials on the Web, and posttest survey), a US $20 gift card for completing the 3-month survey, and a US $35 gift card for completing the 9-month survey. No additional incentives will be provided to participants for completing an MRR form. All incentives will be sent to participants via email.

### Measures

#### Primary Outcomes

Our primary outcomes will include both HPV vaccine initiation (ie, receipt of 1 or more doses of the HPV vaccine series) and completion (ie, receipt of all 3 doses recommended for this age range [13]) during the 9-month follow-up period. The 3- and 9-month surveys will assess how many HPV vaccine doses participants have received. To help maximize the quality of data on our primary outcomes, HPV vaccination status will be confirmed by and based on data from medical records for all participants who complete an MRR form. For those who do not complete an MRR form or who remain unvaccinated during the study, HPV vaccination status will be based on self-reported data from these surveys.

#### Potential Mediators

The results of our pilot test suggest that several theoretically informed constructs may mediate the intervention’s effects on vaccination outcomes [32]. Guided by these previous findings, we will examine several constructs as potential mediators, including *perceived vulnerability* to HPV-related disease (3 items with a 4-point response scale ranging from *no chance* to *high chance*), *perceived severity* of HPV-related disease (3 items with a 4-point response scale ranging from *not at all* to *very*), and *response efficacy* (eg, perceived effectiveness of the HPV vaccine; 2 items with a 5-point response scale ranging from *strongly disagree* to *strongly agree*). Additional constructs will include *rewards of the maladaptive response* (eg, negative social norms of getting the HPV vaccine; 2 items with a 5-point response scale ranging from *strongly disagree* to *strongly agree*), *self-efficacy* to get the HPV vaccine and to talk with a health care provider about the HPV vaccine (2 items with a 5-point response scale ranging from *strongly disagree* to *strongly agree*), *response costs* of getting the HPV vaccine (ie, perceived barriers; 2 items with a 5-point response scale ranging from *strongly disagree* to *strongly agree*), and *intention* to get the HPV vaccine (1 item with a 5-point response scale ranging from *strongly disagree* to *strongly agree*). We will also assess *knowledge* about HPV and the HPV vaccine (7 items with response options of *yes*, *no*, and *I don’t know*), *worry* about getting HPV-related disease (3 items with a 4-point response scale ranging from *not at all* to *a lot*), and *stigma* around HPV (3 items with a 5-point response scale ranging from *strongly disagree* to *strongly agree*). These constructs will be assessed at each survey time point with existing measures and those used in our past studies [17,28,32,46,47].

#### Potential Moderators

The baseline survey will assess demographic and health-related characteristics that we will examine as potential moderators of intervention effects. Demographic characteristics will include participants’ age, race/ethnicity, socioeconomic status, relationship status, sexual orientation (eg, sexual identity, behavior, and attraction), and urbanicity. Health-related characteristics and experiences will include health insurance status, health care utilization, perceived experience of discrimination in receiving health care, disclosure and concealment of sexual orientation to their health care provider, sexual history (eg, number of sexual partners and age at first sexual intercourse), history of HPV-related disease, and HIV status (as some HIV-positive participants may not be aware they can receive the HPV vaccine [13]). The baseline survey will also assess participants’ attitudes about vaccines in general [48] and electronic health literacy [49] (which may affect participants’ understanding and perceptions of the study materials).

#### Acceptability and Additional Survey Measures

The posttest survey will assess acceptability of the Web-based content about the HPV vaccine across the 3 study groups [50]. Survey items will ask about participants’ perceptions of the information viewed (eg, understandability and relevance) and quality of the website (eg, appearance and usability). The 3-month survey will ask participants in the Out-U and Out-I groups about their acceptability of the *Action Plan*, and the 9-month survey will ask these participants about the acceptability of the monthly vaccination reminders (eg, understandability and helpfulness). Study surveys will also examine participants’ communication and information seeking about the HPV vaccine.

#### Engagement

We will measure engagement using administrative data from the project website about study processes and participants’ interaction with study materials (ie, *paradata*). This information will assist in examining whether dosage influences intervention efficacy, which in turn will help inform future dissemination efforts [51]. Paradata will include counts of website log-ins, duration of each log-in, parts of the website visited, duration on each part of the website visited, and functions utilized. For the Out-I group, these data will also include the number and nature of communications that occur during the interactive vaccination reminder process.

### Sample Size and Power

Our target analytic sample size is 1398 YGBMSM (466 per study group). To achieve this analytic sample size, we will randomize a projected 1995 men (665 per study group) and expect 70% retention over the 9-month follow-up period (based on our pilot test [30]). Once randomized, participants who do not complete a given survey can still continue in the study and will be given the opportunity to complete any subsequent surveys. Our target analytic sample size will give us excellent power for making all pairwise comparisons across study groups for our primary outcomes of HPV vaccine initiation and completion, even with a Bonferroni corrected 2-sided alpha of .017 to control the overall type I error rate. For example, assuming an HPV vaccine initiation rate of 20% in the control group, 35% in the Out-U group, and 45% in the Out-I group, we will have 80% power to detect this difference between the Out-U and Out-I groups and more than 99% power to detect this difference between the control and the Out-U groups.

### Statistical Analysis

#### Efficacy

We will examine descriptive statistics for participants’ baseline characteristics and make comparisons across study groups. Using an intention-to-treat approach, we will then use logistic regression models to determine intervention efficacy, with separate models for HPV vaccine initiation and completion. For each outcome, we will perform all pairwise comparisons across study groups. Logistic regression models will produce odds ratios (ORs) and 95% CIs. Sensitivity analyses will include adjusting for possible residual confounders and a tipping point analysis and multiple imputation to examine the impact of any missing outcome data.

#### Mediation

We will examine theoretical constructs as potential mediators using structural equation modeling. We will examine HPV vaccine initiation and completion separately and use a probit link to transform each binary outcome to a standard normal scale [52]. For each outcome, we will fit 2 models using a maximum likelihood approach: a *full* model and a more parsimonious *final* model. The *full* model will include the study group and all theoretical constructs examined. We will assess the overall model fit of the *full* model through the root mean square error of approximation (RMSEA) and other metrics (eg, chi-square test) [53,54]. We will consider an RMSEA of less than 0.07 to indicate an acceptable fit [54]. To create the *final* model, we will remove pathways (ie, associations) from the *full* model based on effect size and the chi-square test for nested models. This process will continue until removing pathways no longer improves the model. The resulting model will be the *final* model. Structural equation models will produce standardized path coefficients that estimate the direct and indirect effects of study group and the theoretical constructs.

#### Moderation

To examine moderation, we will use logistic regression models that include an interaction term between each potential moderator and study group. These logistic regression models will produce ORs and 95% CIs. We will consider moderation to be present if an interaction term has *P*<.05.

## Results

The project has been funded by the National Cancer Institute of the National Institutes of Health under Award Number R37CA226682 (originally awarded as R01CA226682). The institutional review board at The Ohio State University has approved this study (study number 2019C0028). The Ohio State University served as the reviewing institution with other study sites ceding review. We have also registered this clinical trial (NCT04032106). Recruitment and enrollment for the RCT began in Fall 2019.

## Discussion

There are several potential challenges and limitations to our forthcoming RCT. First, although we will confirm participants’ HPV vaccination status through medical records when possible for primary outcomes, we will use self-reported data for participants who do not provide a signed MRR. We will also rely on the participants’ self-reported HPV vaccination status in determining study eligibility. However, previous research suggests that most young adults can accurately report their HPV vaccination status, with only a 2.2% net bias in self-reported HPV vaccination data among this age group [55]. Second, as we attempt to recruit a large sample size of YGBMSM and follow up with participants for 9 months, it is possible that we may encounter challenges with our recruitment and retention goals. To address this, we will recruit participants through multiple social media sites, aid both recruitment and retention by offering incentives, and further aid retention by using automated notifications for the completion of follow-up surveys. Third, a concern for Web-based health research is the presence of fraudulent accounts (eg, users with multiple accounts). We will use several recommended strategies [56] for minimizing this concern, including inspecting account information for similarities between accounts, requiring potential participants to verify their text message number as part of the project account setup process (and not allowing the same text message number to be used for multiple accounts), inspecting survey data for illogical responses, and providing incentives only after study activities are completed. We will review new accounts on a regular basis during recruitment to monitor for fraudulent accounts, and accounts suspected of being fraudulent will be deactivated immediately. Fourth, we acknowledge the chance of contamination occurring in health behavior interventions. We believe the chance for contamination is low for this study because we will recruit throughout the United States, the project website will require log-in, and participants will receive only the study materials for their randomized study arm. Finally, this RCT will focus on cisgender males, but if the intervention is shown to be efficacious, it will be important for future efforts to also include transgender individuals.

The goal of *Outsmart HPV* is to increase HPV vaccine coverage among YGBMSM. This study is a critical step toward achieving this goal because it will comprehensively evaluate *Outsmart HPV*. The results will provide an understanding of the intervention’s efficacy (including any potential differences between the use of unidirectional and interactive vaccination reminders), mediators, and moderators. If proven efficacious, *Outsmart HPV* has the potential to fill an important gap by providing a Web-based intervention that allows YGBMSM to learn about HPV vaccination and promotes vaccination.

## Appendix

Multimedia Appendix 1 Peer-reviewer report from the National Institutes of Health.

## Abbreviations

ACIP: Advisory Committee on Immunization Practices
GBMSM: gay, bisexual, and other men who have sex with men
GIF: Graphics Interchange Format
HPV: human papillomavirus
MRR: medical record release
OR: odds ratio
Out-I group: Outsmart HPV that includes monthly interactive vaccination reminders
Out-U group: Outsmart HPV with monthly unidirectional vaccination reminders
RCT: randomized controlled trial
RMSEA: root mean square error of approximation
VIS: Vaccine Information Statement
YGBMSM: young gay, bisexual, and other men who have sex with men
