# Supplementary material for: A Web-Based Human Papillomavirus Vaccination Intervention for Young Gay, Bisexual, and Other Men Who Have Sex With Men: Protocol for a Randomized Controlled Trial
Source: JMIR Res Protoc. 2020 Feb 24;9(2):e16294. doi: 10.2196/16294 (PMC7063529; doi:10.2196/16294)
Supplement: Multimedia Appendix 1 [file resprot_v9i2e16294_app1.pdf]

SUMMARY STATEMENT
(Privileged Communication)

PROGRAM CONTACT:
Vaurice Starks
301-402-9375
starksv@mail.nih.gov

Release Date: **10/27/2017**
Revised Date:

Application Number: **1 R01 CA226682-01**

Principal Investigators (Listed Alphabetically):

MCREE, ANNIE-LAURIE
REITER, PAUL L (Contact)

Applicant Organization: OHIO STATE UNIVERSITY

Review Group: **HDEP**
Health Disparities and Equity Promotion Study Section

Meeting Date: **10/12/2017**
Council: **JAN 2018**
Requested Start: **04/01/2018**

RFA/PA: **PA15-261**
PCC: **7LER**
Dual IC(s): **MD**

Project Title: **A Randomized Controlled Trial of an HPV Vaccine Intervention for Young Sexual Minority Men**
SRG Action: **Impact Score:19 Percentile:6**
Next Steps: **Visit https://grants.nih.gov/grants/next_steps.htm**
Human Subjects: **30-Human subjects involved - Certified, no SRG concerns**
Animal Subjects: **10-No live vertebrate animals involved for competing appl.**
Gender: **3A-Only men, scientifically acceptable**
Minority: **1A-Minorities and non-minorities, scientifically acceptable**
Children: **3A-No children included, scientifically acceptable**
NIH Defined Phase III Clinical trial

Project Year	Direct Costs Requested	Estimated Total Cost
1	434,566	686,020
2	476,227	751,787
3	443,675	700,400
4	425,384	671,525
TOTAL	1,779,852	2,809,732

ADMINISTRATIVE BUDGET NOTE: The budget shown is the requested budget and has not been adjusted to reflect any recommendations made by reviewers. If an award is planned, the costs will be calculated by Institute grants management staff based on the recommendations outlined below in the COMMITTEE BUDGET RECOMMENDATIONS section.
EARLY STAGE INVESTIGATOR
NEW INVESTIGATOR

1R01CA226682-01 Reiter, Paul

EARLY STAGE INVESTIGATOR
NEW INVESTIGATOR

SCIENTIFIC REVIEW OFFICER'S NOTES

RESUME AND SUMMARY OF DISCUSSION: This application proposes to evaluate the efficacy of a mobile health HPV vaccine intervention using unidirectional and interactive HPV vaccine reminders on HPV vaccine initiation and completion for young sexual minority men (YSMM). If successful, findings from this study may inform interventions targeting HPV in young sexual minority males. This is a highly significant area of inquiry. The investigative team has considerable experience with strong evidence of prior collaboration in an excellent environment. The reviewers noted several additional strengths in the application including the significance of the public health issue addressed with a strong scientific premise for HPV risks, innovative comparison of unidirectional and interactive HPV vaccine reminders, strong pilot data, and the rigorous randomized study design appropriately guided by sound theoretical approaches. The reviewers noted a few minor weaknesses including the justification for social media inclusion criteria and the rationale to aggregate MSM despite potential differences in risk profiles. Although the investigative team has a considerable track record of prior work in the target population, some reviewers raised concerns about the feasibility of participant recruitment. There is a missed opportunity in the study design to explore the intersectionality of identity for gay-identified bisexual and non-heterosexual men. Overall, the strengths outweighed minor weaknesses noted in the approach and the panel agreed that the findings are expected to have a high impact on HPV vaccination interventions for YSMM.

DESCRIPTION (provided by applicant): Our overall goal is to increase human papillomavirus (HPV) vaccination among young sexual minority men (YSMM). Sexual minority men (i.e., men who have sex with men or identify as gay or bisexual) have high rates of HPV infection and HPV-related disease, including anal cancer. HPV vaccine is recommended for sexual minority men through age 26, yet fewer than 21% of YSMM ages 18-26 have received any HPV vaccine doses (i.e., initiation) and fewer than 10% have received all three recommended doses (i.e., completion). To address this issue, we recently developed and pilot tested a theoretically-informed mobile health HPV vaccine intervention for YSMM (Outsmart HPV). To our knowledge, Outsmart HPV is the first HPV vaccine intervention developed for this population. The pilot study established the intervention's feasibility and acceptability and produced very promising preliminary data. However, the pilot nature of this study precluded us from formally establishing intervention efficacy or examining additional intervention mechanism processes. The proposed study is a critical next step in this line of research because it will build upon our pilot study to comprehensively evaluate Outsmart HPV via a well-powered randomized controlled trial. We will recruit 1995 unvaccinated YSMM ages 18-25 from the US via social media and randomize each participant to either: a) standard information about HPV and HPV vaccine via a mobile-friendly website (control group); b) Outsmart HPV with unidirectional vaccine reminders; or c) Outsmart HPV with interactive vaccine reminders. Aim 1 will determine the efficacy of Outsmart HPV on increasing HPV vaccine initiation and completion. In doing so, the aim will determine which type of HPV vaccine reminders (unidirectional or interactive) most effectively increases vaccination as part of the Outsmart HPV intervention. Aim 2 will identify mediators that explain the relationship between study arm and HPV vaccine initiation and completion. This will identify the mechanism by which the intervention affects HPV vaccination (i.e., how the intervention leads to changes in theoretical constructs which in turn lead to vaccination). Aim 3 will determine if intervention efficacy differs across key demographic and health-related characteristics of participants (i.e., moderation). This will allow us to determine if Outsmart HPV has differential efficacy across subgroups and, if so, to identify those men for whom the intervention may require further adaptation in the future. Results of the proposed study will provide an evidence base regarding intervention efficacy, mediators, and moderators. These findings will offer a

sophisticated understanding of Outsmart HPV that is needed to effectively and efficiently disseminate this innovative intervention to YSMM in the future.

PUBLIC HEALTH RELEVANCE: The proposed study will comprehensively evaluate Outsmart HPV, an innovative mobile health HPV vaccine intervention that was recently developed for young sexual minority men. In doing so, the proposed study will provide an evidence base regarding the intervention's efficacy, mediators, and moderators. Results will provide a sophisticated understanding of the intervention that is needed to effectively and efficiently disseminate the intervention in the future.

CRITIQUE 2

Significance: 1
Investigator(s): 1
Innovation: 1
Approach: 2
Environment: 1

Overall Impact: This outstanding application seeks to test the efficacy of a theory-based, targeted and tailored, mobile health HPV vaccine intervention ("Outsmart HPV") for young sexual minority men (YSMM) over a 4 year timeline. The intervention will be tested in a 3-arm RCT that is powered to detect differences in initiation and completion rates and includes comparison with an appropriate control arm as well as an arm that is responsive to emerging science in the area of mHealth. Additionally, the trial is adequately powered to identify theory-based mediators and demographic moderators of the intervention effect, which will provide needed evidence for dissemination efforts. The intervention targets a significant population: at-risk young men who are vulnerable to disparities in HPV vaccination and HPV-related diseases, including several cancers for which routine screening is not available. The proposed trial is rigorously designed, the intervention elements are reproducible and well-justified based on health behavior theory, and the underlying premise for the work is supported by strong preliminary data from a feasibility study conducted by the team in preparation for the randomized trial. The investigative team is exceptionally and uniquely well-qualified to conduct the study, with a strong MPI plan and clear contributions made by nationally recognized experts in men's reproductive health and HPV vaccination through the inclusion of an Expert Advisory Board. The environment and resources available will ensure success with each critical element of this trial, from enrollment to intervention delivery, analysis, and dissemination and implementation planning. The strengths of this application far outweigh a few minor weaknesses noted in the approach.

1. Significance:
Strengths
- Preliminary results support the feasibility of the Outsmart HPV intervention among YSMM and provide sound, relevant estimates for planning the proposed trial
- Interventions for HPV vaccination have not targeted YSMM, who are at high risk of HPV-related diseases and less aware of HPV vaccination, thus, a successful intervention with understanding of mediators and moderators of intervention efficacy would have a high impact on the field of male sexual minority health

Weaknesses
- None noted.

2. Investigator(s):
Strengths
- Highly accomplished investigative team containing all of the necessary expertise to conduct the proposed work, including behavioral interventions, HPV vaccination, YSMM health, medicine, mHealth/health communication, and biostatistics

- Drs. Reiter and McRee collaborated successfully on the feasibility study (NCI R21) for the Outsmart HPV intervention, have numerous co-authored papers, and a detailed MPI Leadership Plan
- An Expert Advisory Board will be actively engaged with the investigators throughout the duration of the project with meaningful and specific responsibilities to enhance the rigor of the study; of note exceptional relevant expertise includes Drs. Zimet and Bell who have provided letters of support

Weaknesses

- None noted.

3. Innovation:

Strengths

- Interventions specifically targeting HPV vaccination among YSMM have not been developed
- Conducting an appropriately powered comparison of unidirectional vs. interactive vaccine reminders in a randomized design is novel, responsive to recent evidence, and would inform the field of mHealth research more generally

Weaknesses

- None noted.

4. Approach:

Strengths

- Strong preliminary data support the feasibility of the Outsmart HPV intervention; the underlying rationale for the intervention components is well-justified by 3 relevant theoretical/conceptual frameworks (PMT, IMB, MSM)
- Trial is adequately (>80%) powered to determine efficacy (initiation and completion), identify theory-based mediators, and evaluate effect-modifiers of the intervention (demographic and health-related)
- Intervention content and recruitment methods will be finalized in response to sequential qualitative input from YSMM via online focus groups
- Two intervention arms have a strong scientific rationale based in mHealth literature (interactive vs. unidirectional reminders), control arm offers access to VIS (standard-of-care HPV vaccine materials from CDC)
- Rigorous development of interactive response message library ensures accurate and "standard" replies are given in response to participant needs

Weaknesses

- Recruitment methods will exclude YSMM who are not engaged with social media; this is a minor weakness, as it will be a small minority of individuals who will likely not be responsive to mHealth approaches and require other strategies for reach/intervention
- Vaccination records (signature of release of medical information) will not be required for study participation which could threaten the validity of self-reported outcome data

5. Environment:

Strengths

- The Ohio State University provides outstanding physical, human, and intellectual resources to the investigative team including secure storage, grants administration, human subjects review, and resources inherent to having a Comprehensive Cancer Center
- Technologies needed to carry out the research are available through itracks (online focus groups) and the Center for Health Communications Research at the University of Michigan (mHealth intervention)

Weaknesses

- None noted

Protections for Human Subjects:
Acceptable Risks and/or Adequate Protections

- Adequate protections are outlined for the confidentiality of the data and the safety of the subjects; informed consent is appropriate; authorization for release of HPV vaccination data is voluntary; staff will complete GCP training

Data and Safety Monitoring Plan (Applicable for Clinical Trials Only):
Acceptable

- An adequate plan is provided to regularly monitor the integrity of the data and the safety of the subjects; appropriate and timely reporting of adverse events, data breaches, and protocol deviations is described, a board seems unnecessary.

Inclusion of Women, Minorities and Children:

- Sex/Gender: Distribution justified scientifically
- Race/Ethnicity: Distribution justified scientifically
- For NIH-Defined Phase III trials, Plans for valid design and analysis: Scientifically acceptable
- Inclusion/Exclusion of Children under 18: Excluding ages <18; justified scientifically
- Only males, includes minorities and non-minorities, excluding children. Intervention targeted to sexual minority (gay, bisexual) males; 18 and older averts the need for parental permission/involvement in a study that reveals sexual minority status - this adds an element of safety and reflects data showing YSMM may not disclose their sexual orientation prior to age 18

Vertebrate Animals:
Not Applicable (No Vertebrate Animals)

Biohazards:
Not Applicable (No Biohazards)

Select Agents:
Not Applicable (No Select Agents)

Resource Sharing Plans:
Acceptable

Authentication of Key Biological and/or Chemical Resources:
Not Applicable (No Relevant Resources)

Budget and Period of Support:
Recommend as Requested

CRITIQUE 2

Significance: 2
Investigator(s): 1
Innovation: 2
Approach: 2
Environment: 1

Overall Impact: Overall this application has several strengths. This application addresses YSMM, an understudied population that faces greater disease burden and disparities from HPV related cancers. It

has a strong team of investigators with the appropriate expertise and relevant experience to conduct the project. And finally, the scientific premise is clear. Aims and hypotheses are clearly articulated and justified. Minor weaknesses that dampen enthusiasm for this application include that the project includes both a unidirectional and interactive condition that inflates the sample size needed to conduct a fully powered test of the intervention. Efficacy was established in the pilot for unidirectional messages; it is unclear why the interactive component needs a different intervention group since it includes the unidirectional message and then provides additional intervention. The investigators do not use statistical controls to account for the variance contributed by the interactive messages. Then the required sample size would be more manageable. The recruitment plan does not appear feasible given the team's own experience with this population. Finally, additional information is needed on the power analysis. The investigators note that "projections are based on pilot results (with slight increases)" but we do not know how "slight." These weaknesses dampen enthusiasm for an otherwise impactful application.

1. Significance:
Strengths
- This application addresses YSMM an understudied population that faces greater disease burden and disparities from HPV related cancers
- CDC supports the need for catch up vaccines in this population through age 26
- The application is built on a strong scientific premise

Weaknesses
- The investigators should justify the need to examine unidirectional versus interactive messages.

2. Investigator(s):
Strengths
- Strong team of investigator with appropriate expertise and relevant experience to conduct the project.

Weaknesses
- None noted.

3. Innovation:
Strengths
- This a multicomponent intervention targeting all steps need to complete the HPV vaccination process: information, planning and follow-up
- The intervention will help participants locate a provider in order to obtain vaccination.
- This project will develop an intervention for YSMM, a population that will benefit from increased vaccine coverage.

Weaknesses
- None noted

4. Approach:
Strengths
- The intervention is based on strong preliminary evidence of intervention efficacy from a previous pilot study.
- Phase I and Phase II are clearly delineated. The project has well articulated aims and testable hypotheses.
- The scientific premise is clear.

Weaknesses
- Efficacy was established in the pilot for unidirectional messages; it is unclear why the interactive component needs a different intervention group since it includes the unidirectional message and then provides additional intervention. It is unclear if the investigators are suggesting that adding the interactive component to the existing intervention changes it significantly from the pilot. The

recruitment plan does not appear feasibly given the teams own experience with this population. In 7 months, the team recruited 150 study participants (however in 2.5 years they plan to recruit 1995)

- The investigators state that their desired sample size is 1398, however, it is not clear what the sample sized based on. Additional information on the power analysis is needed. The investigators note that "projections are based on pilot results (with slight increases..)" but we do not know how "slight"

5. Environment:
Strengths
- Environment appears adequate to conduct the proposed work.
- The team has a strong track record of collaboration with the Principal Investigator

Weaknesses
- None noted.

Protections for Human Subjects:
Acceptable Risks and/or Adequate Protections
- Risks and protections were adequately justified.

Data and Safety Monitoring Plan (Applicable for Clinical Trials Only):
Acceptable
  - Well articulated plan

Inclusion of Women, Minorities and Children:
- Sex/Gender: Distribution justified scientifically
- Race/Ethnicity: Distribution justified scientifically
- For NIH-Defined Phase III trials, Plans for valid design and analysis: Scientifically acceptable
- Inclusion/Exclusion of Children under 18: Excluding ages <18; justified scientifically
- The application is focused on catch up vaccines.

Vertebrate Animals:
Not Applicable (No Vertebrate Animals)

Biohazards:
Not Applicable (No Biohazards)

Select Agents:
Not Applicable (No Select Agents)

Resource Sharing Plans:
Not Applicable (No Relevant Resources)

Authentication of Key Biological and/or Chemical Resources:
Not Applicable (No Relevant Resources)

Budget and Period of Support:
Recommend as Requested

CRITIQUE 3

Significance: 1
Investigator(s): 1

Innovation: 2
Approach: 2
Environment: 1

Overall Impact: Increasing uptake of HPV vaccinations is an important public health concern. The application has a number of important strengths including: the focus on YMSM; addressing an important cancer risk reduction behavior; guided by sound theoretical approaches; based on strong preliminary data; and rigorous 3 group design. This is an experienced team with a demonstrated ability to recruit and retain the target population. Other strengths include objective measures of vaccine uptake and clear analysis plan. There are weaknesses that dampen enthusiasm including the collapsing YMSM into a single category. Research shows differences in risk profiles based on identity status. The investigators should consider examining by sexual identity – gay, bisexual, non-heterosexual and behavior dimensions to address the weaknesses.

1. Significance:
Strengths
- Focus on males and uptake of HPV vaccination among a high risk group of YMSM
- Addressing an important cancer risk reduction behavior
- Based on a strong scientific premise

Weaknesses
- None noted

2. Investigator(s):
Strengths
- This is a strong investigative team with the relevant expertise and experience to complete the research.
- Strong letters of collaboration
- Productive funding and dissemination track record

Weaknesses
- None

3. Innovation:
Strengths
- Use technology-based methods for recruitment and implementation
- Compare unidirectional and interactive HPV vaccine reminders

Weaknesses
- None

4. Approach:
Strengths
- Based on strong preliminary data
- Demonstrated ability to recruit and retain target population
- Rigorous scientific design
- Objective measures of vaccine uptake
- Clear analysis plan

Weaknesses
- Collapsing YMSM into a single category. Research shows differences in risk profiles based on identity status. Should consider examining by sexual identity – gay, bisexual, non-heterosexual and behavior dimensions.

5. Environment:

Strengths
- The environment and institutional resources are appropriate for the conduct of the study

Weaknesses
- None noted

Protections for Human Subjects:
Acceptable Risks and/or Adequate Protections
- Not applicable

Data and Safety Monitoring Plan (Applicable for Clinical Trials Only):
Not Applicable (No Clinical Trials)
  - Not applicable

Inclusion of Women, Minorities and Children:
- Sex/Gender: Distribution justified scientifically
- Race/Ethnicity: Distribution justified scientifically
- For NIH-Defined Phase III trials, Plans for valid design and analysis: Not applicable
- Inclusion/Exclusion of Children under 18: Excluding ages <18; justified scientifically

Vertebrate Animals:
Not Applicable (No Vertebrate Animals)

Biohazards:
Not Applicable (No Biohazards)

Select Agents:
Not Applicable (No Select Agents)

Resource Sharing Plans:
Not Applicable (No Relevant Resources)

Authentication of Key Biological and/or Chemical Resources:
Not Applicable (No Relevant Resources)

Budget and Period of Support:
Recommend as Requested

THE FOLLOWING SECTIONS WERE PREPARED BY THE SCIENTIFIC REVIEW OFFICER TO SUMMARIZE THE OUTCOME OF DISCUSSIONS OF THE REVIEW COMMITTEE, OR REVIEWERS' WRITTEN CRITIQUES, ON THE FOLLOWING ISSUES:

PROTECTION OF HUMAN SUBJECTS: ACCEPTABLE

INCLUSION OF WOMEN PLAN: ACCEPTABLE

INCLUSION OF MINORITIES PLAN: ACCEPTABLE

INCLUSION OF CHILDREN PLAN: ACCEPTABLE

COMMITTEE BUDGET RECOMMENDATIONS: The budget was recommended as requested.

SCIENTIFIC REVIEW OFFICER'S NOTES:

The NIH special practice for new investigator R01 applications reviewed in the Center for Scientific Review study sections applies to this application. Resubmission (amended -A1) R01 applications from new investigators may be submitted on a special receipt date for review in the very next review cycle. See this notice in the NIH Guide for Grants and Contracts for more details: http://grants1.nih.gov/grants/guide/notice-files/NOT-OD-11-057.html.

You should contact the NIH program officer whose name is shown in the upper left hand corner of page one of this Summary Statement for information about whether this application may be fundable or whether you will need to submit an amended application. The program officer can also help you decide whether the changes and improvements necessary to address the weaknesses noted in the reviewers' critiques could be accomplished in the relatively short time available. You are also strongly advised to seek input from mentors, your Department chair, etc.

If you choose to submit a resubmission application for the next review cycle under this policy for new investigators, your amended application must be received at NIH no later than Monday, December 11, 2017.

You may, of course, choose to take more time to resubmit your application. If so, you should prepare the resubmission for the normal dates for amended applications as specified in this table: http://grants1.nih.gov/grants/funding/submissionschedule.htm.

Footnotes for 1 R01 CA226682-01; PI Name: Reiter, Paul L

NIH has modified its policy regarding the receipt of resubmissions (amended applications). See Guide Notice NOT-OD-14-074 at http://grants.nih.gov/grants/guide/notice-files/NOT-OD-14-074.html. The impact/priority score is calculated after discussion of an application by averaging the overall scores (1-9) given by all voting reviewers on the committee and multiplying by 10. The criterion scores are submitted prior to the meeting by the individual reviewers assigned to an application, and are not discussed specifically at the review meeting or calculated into the overall impact score. Some applications also receive a percentile ranking. For details on the review process, see http://grants.nih.gov/grants/peer_review_process.htm#scoring.

MEETING ROSTER

Health Disparities and Equity Promotion Study Section
Healthcare Delivery and Methodologies Integrated Review Group
CENTER FOR SCIENTIFIC REVIEW
HDEP
10/12/2017 - 10/13/2017

Notice of NIH Policy to All Applicants: Meeting rosters are provided for information purposes only. Applicant investigators and institutional officials must not communicate directly with study section members about an application before or after the review. Failure to observe this policy will create a serious breach of integrity in the peer review process, and may lead to actions outlined in NOT-OD-14-073 at https://grants.nih.gov/grants/guide/notice-files/NOT-OD-14-073.html and NOT-OD-15-106 at https://grants.nih.gov/grants/guide/notice-files/NOT-OD-15-106.html, including removal of the application from immediate review.

CHAIRPERSON(S)

MATTHEWS, ALICIA K, PHD
PROFESSOR
DEPARTMENT OF HEALTH SYSTEMS SCIENCES
COLLEGE OF NURSING
UNIVERSITY OF ILLINOIS AT CHICAGO
CHICAGO, IL 60612

MEMBERS

ALLEN, HEIDI LYNN, PHD *
SCHOOL OF SOCIAL WORK
COLUMBIA UNIVERSITY
NEW YORK, NY 10027

ANTHONY, RENAISA SPRING, MD *
DEPUTY DIRECTOR
CENTER FOR REDUCING HEALTH DISPARITIES
COLLEGE OF PUBLIC HEALTH
UNIVERSITY OF NEBRASKA MEDICAL CENTER
OMAHA, NE 68198

ARRIOLA, KIMBERLY, PHD
PROFESSOR
DEPARTMENT OF BEHAVIORAL SCIENCES AND
HEALTH EDUCATION
ROLLINS SCHOOL OF PUBLIC HEALTH OF
EMORY UNIVERSITY
ATLANTA, GA 30322

CABASSA, LEOPOLDO J, PHD
ASSOCIATE PROFESSOR
SCHOOL OF SOCIAL WORK
COLUMBIA UNIVERSITY
NEW YORK, NY 10032

CRESPO, CARLOS J, DRPH
ASSOCIATE DEAN
OREGON HEALTH AND SCIENCE UNIVERSITY
PORTLAND STATE UNIVERSITY
SCHOOL OF PUBLIC HEALTH
PORTLAND, OR 97207

DASH, CHIRANJEEV, MBBS, PHD *
ASSOCIATE PROFESSOR
DEPARTMENT OF ONCOLOGY
GEORGETOWN LOMBAREB COMPREHENSIVE
CANCER CENTER
GEORGETOWN UNIVERSITY
WASHINGTON, DC 20057

GARCIA, VICTOR Q, PHD *
PROFESSOR AND DIRECTOR
MID-ATLANTIC RESEARCH AND
TRAINING INSTITUTE (MARTI)
INDIANA UNIVERSITY OF PENNSYLVANIA
INDIANA, PA 15701

GUERRERO, ERICK, PHD *
ASSOCIATE PROFESSOR
SCHOOL OF SOCIAL WORK
UNIVERSITY OF SOUTHERN CALIFORNIA
LOS ANGELES, CA 90089

HUEBNER, DAVID M, PHD *
ASSOCIATE PROFESSOR
DEPARTMENT OF PREVENTION AND COMMUNITY HEALTH
MILKEN INSTITUTE
SCHOOL OF PUBLIC HEALTH
THE GEORGE WASHINGTON UNIVERSITY
WASHINGTON, DC 20052

KAHOLOKULA, JOSEPH KEAWE'AIMOKU, PHD
PROFESSOR
DEPARTMENT OF NATIVE HAWAIIAN HEALTH
UNIVERSITY OF HAWAII AT MANOA
HONOLULU, HI 96813

KARNIK, NIRANJAN, MD, PHD
ASSOCIATE PROFESSOR
DEPARTMENT OF PSYCHIATRY
RUSH UNIVERSITY MEDICAL CENTER
CHICAGO, IL 60612

MARSHAL, MICHAEL P, PHD
ASSOCIATE PROFESSOR
DEPARTMENTS OF PSYCHIATRY AND PEDIATRICS
SCHOOL OF MEDICINE
UNIVERSITY OF PITTSBURGH
PITTSBURGH, PA 15213

MUNSON, MICHELLE R, PHD *
ASSOCIATE PROFESSOR
SILVER SCHOOL OF SOCIAL WORK
NEW YORK UNIVERSITY
NEW YORK, NY 10003

MURILLO, ROSENDA, PHD *
ASSISTANT PROFESSOR
DEPARTMENT OF PSYCHOLOGICAL HEALTH AND
LEARNING SCIENCES
UNIVERSITY OF HOUSTON
HOUSTON, TX 77204-5029

NUNEZ-SMITH, MARCELLA, MD
ASSOCIATE PROFESSOR OF MEDICINE AND PUBLIC
HEALTH
YALE UNIVERSITY SCHOOL OF MEDICINE
NEW HAVEN, CT 06520

OSYPUK, THERESA LOUISE, SCD *
ASSOCIATE PROFESSOR
SCHOOL OF PUBLIC HEALTH
UNIVERSITY OF MINNESOTA
MINNEAPOLIS, MN 55454

PEARMAN, TIMOTHY PATRICK, PHD *
PROFESSOR, DIRECTOR SUPPORTIVE ONOLOGY
LURIE COMPREHENSIVE CANCER CENTER
NORTHWESTERN UNIVERSITY SCHOOL OF MEDICINE
CHICAGO, IL 60611

POLIVKA, BARBARA J, PHD *
SHIRLEY B POWERS ENDOWED CHAIR AND PROFESSOR
UNIVERSITY OF LOUISVILLE SCHOOL OF NURSING
NORTON HEALTHCARE
LOUISVILLE, KY 40202

POLLACK, CRAIG EVAN, MD *
ASSOCIATE PROFESSOR OF MEDICINE
DIVISION OF GENERAL INTERNAL MEDICINE
JOHNS HOPKINS SCHOOL OF MEDICINE
JOHNS HOPKINS UNIVERSITY
BALTIMORE, MD 21218

POSTON, WALKER S, PHD *
DIRECTOR AND SENIOR PRINCIPAL INVESTIGATOR
INSTITUTE FOR BIOBEHAVIORAL HEALTH RESEARCH
CENTER FOR FIRE, RESCUE AND EMS HEALTH RESEARCH
NATIONAL DEVELOPMENT
AND RESEARCH INSTITUTES NDRI
NEW YORK, NY 10010

QUINN, GWENDOLYN P, PHD
PROFESSOR
DEPARTMENT OF ONCOLOGIC SCIENCES
H LEE MOFFITT CANCER CENTER AND RESEARCH
INSTITUTE
COLLEGE OF MEDICINE
UNIVERSITY OF SOUTH FLORIDA
TAMPA, FL 33612

RADECKI BREITKOPF, CARMEN, PHD
ASSOCIATE PROFESSOR
DEPARTMENT OF HEALTH SCIENCES RESEARCH
DIVISION OF HEALTH CARE POLICY AND RESEARCH
MAYO CLINIC COLLEGE OF MEDICINE
ROCHESTER, MN 55905

SARPONG, DANIEL F, PHD *
DIRECTOR AND ENDOWED CHAIR
DEPARTMENT OF PHARMACY
XAVIER UNIVERSITY
NEW ORLEANS, LA 70125

SEGUIN, REBECCA ANNE, PHD *
ASSOCIATE PROFESSOR
DIVISION OF NUTRITIONAL SCIENCES
CORNELL UNIVERSITY
ITHACA, NY 14853

SHUMWAY, MARTHA, PHD
ASSOCIATE PROFESSOR
DEPARTMENT OF PSYCHIATRY
SCHOOL OF MEDICINE
UNIVERSITY OF CALIFORNIA SAN FRANCISCO
SAN FRANCISCO, CA 94143

SINGH, PRAMIL NAND, DRPH *
DIRECTOR AND ASSOCIATE PROFESSOR
CENTER FOR HEALTH RESEARCH
LOMA LINDA UNIVERSITY
LOMA LINDA, CA 92350

SKOLARUS, LESLI ELIZABETH, MD *
ASSOCIATE PROFESSOR
CARDIOVASCULAR CENTER
UNIVERSITY OF MICHIGAN
ANN ARBOR, MI 48109

SOULAKOVA, JULIA, PHD *
ASSOCIATE PROFESSOR
BURNETT SCHOOL OF BIOMEDICAL SCIENCES
UNIVERSITY OF CENTRAL FLORIDA
ORLANDO, FL 32827

THOMAS, STEPHEN B, PHD
DIRECTOR, MARYLAND CENTER FOR HEALTH EQUITY
PROFESSOR, DEPARTMENT OF HEALTH SERVICES
ADMINISTRATION, SCHOOL OF PUBLIC HEALTH
UNIVERSITY OF MARYLAND, COLLEGE PARK
COLLEGE PARK, MD 20742

THORPE, ROLAND J JR, PHD *
ASSOCIATE PROFESSOR
JOHN HOPKINS BLOOMBERG SCHOOL OF PUBLIC HEALTH
DEPARTMENT OF HEALTH POLICY AND MANAGEMENT
HOPKINS CENTER FOR HEALTH DISPARITIES SOLUTIONS
BALTIMORE, MD 21205

WHITEHEAD, NICOLE ENNIS, PHD *
ASSISTANT PROFESSOR
DEPARTMENT OF CLINICAL AND HEALTH PSYCHOLOGY
COLLEGE OF PUBLIC HEALTH AND HEALTH PROFESSIONS
GAINESVILLE, FL 32610

WU, BEI, PHD
PROFESSOR AND DEAN'S CHAIR
RORY MEYERS COLLEGE OF NURSING
NEW YORK UNIVESITY
NEW YORK , NY 10010

MAIL REVIEWER(S)

KLASSEN, ANN CARROLL, PHD
PROFESSOR
DEPARTMENT OF COMMUNITY HEALTH AND PREVENTION
SCHOOL OF PUBLIC HEALTH
DREXEL UNIVERSITY
PHILADELPHIA, PA 19104

SHALOWITZ, MADELEINE ULLMAN, MD
DIRECTOR OF BIOMEDICAL RESEARCH INFORMATICS
ASSOCIATE PROFESSOR
DEPARTMENT OF PEDIATRICS
UNIVERSITY OF CHICAGO
EVANSTON, IL 60201

SCIENTIFIC REVIEW OFFICER

BELLINGER, JESSICA, PHD
SCIENTIFIC REVIEW ADMINISTRATOR
CENTER FOR SCIENTIFIC OF REVIEW
NATIONAL INSTITUTES OF HEALTH
BETHESDA, MD 20892

EXTRAMURAL SUPPORT ASSISTANT

BUTLER, SEAN
LEAD - EXTRAMURAL SUPPORT ASSISTANT
CENTER FOR SCIENTIFIC REVIEW
NATIONAL INSTITUTE OF HEALTH
BETHESDA, MD 20892

* Temporary Member. For grant applications, temporary members may participate in the entire meeting or may review only selected applications as needed.

Consultants are required to absent themselves from the room during the review of any application if their presence would constitute or appear to constitute a conflict of interest.
